# Urinary tract infection (UTI) and infection control in municipal nursing homes

**DOI:** 10.1186/1753-6561-5-S6-P161

**Published:** 2011-06-29

**Authors:** A-M Andersen, B Kristensen

**Affiliations:** 1 Clinical Microbiology, Aarhus University Hospital, Skejby, Århus N, Denmark

## Introduction / objectives

A formalized infection control organization does not exist in the Danish municipal sector. The prevalence of infections among residents in nursing homes is unknown and knowledge is sparse on the connection between primary care staff knowledge and infection rates.

## Methods

This study investigated educational level and infection control knowledge as well as direct observations of hygiene performance among staff in four selected nursing homes. Data were collected through individual, self-administered questionnaires filled in by 70 staff members. Moreover, data were collected on infection rates (UTIs) between 1 January and 31 December 2009 in a cohort of all 2761 nursing home residents in the Municipality of Aarhus in Denmark (310.653 inhabitants). Data were collected from medical databases on infection rates and antibiotic use.

## Results

Handhygiene compliance (alcohol-based handrubbing) among staff-members was 52.6 % before a procedure, 59.6 % after a procedure and the infection control knowledge was deemed insufficient among the healthcare staff. 26 (37.1%) of the 70 participants did not have any infection control education. During 2009, 961 (34.8 %) of nursing home residents (247 men and 714 women) had at least one UTI. A total of 861 (31.2 %) residents were treated with UTI specific antibiotics by their GP at least once during 2009.

## Conclusion

UTIs are common among nursing home residents, and antibiotic use is very high.Infection control knowledge and practices among nursing home staff were insufficient. Further studies are needed to shed light on the association between infection rates and infection control efforts at individual nursing homes.

## Disclosure of interest

None declared.

